# Genetics and geometry of canalization and developmental stability in *Drosophila subobscura*

**DOI:** 10.1186/1471-2148-5-7

**Published:** 2005-01-22

**Authors:** Mauro Santos, Pedro Fernández Iriarte, Walkiria Céspedes

**Affiliations:** 1 Grup de Biologia Evolutiva (GBE), Departament de Genètica i de Microbiologia, Universitat Autònoma de Barcelona, 08193 Bellaterra (Barcelona), Spain; 2 CICyTTP-CONICET, Matteri y España (3105) Diamante, Entre Ríos, Argentina

## Abstract

**Background:**

Many properties of organisms show great robustness against genetic and environmental perturbations. The terms canalization and developmental stability were originally proposed to describe the ability of an organism to resist perturbations and to produce a predictable target phenotype regardless of random developmental noise. However, the extent to which canalization and developmental stability are controlled by the same set of genes and share underlying regulatory mechanisms is largely unresolved.

**Results:**

We have analyzed the effects of clinal genetic variation (inversion polymorphism) on wing asymmetry by applying the methods of geometric morphometrics in the context of quantitative genetics using isochromosomal lines of *Drosophila subobscura*. For the analysis of overall size, developmental stability was positively correlated with levels of heterozygosity and development at the optimal temperature. For analyses of shape, the overall comparisons by matrix correlations indicate that inter- and intraindividual variation levels were poorly correlated, a result also supported when comparing the vectors describing patterns of variation of landmark position. The lack of similarity was basically due to the discrepancy between the genetic and environmental components of the interindividual variation. Finally, the analyses have also underscored the presence of genetic variation for directional asymmetry.

**Conclusions:**

The results strongly support the hypothesis that environmental canalization and developmental stability share underlying regulatory mechanisms, but environmental and genetic canalization are not functionally the same. A likely explanation for this lack of association is that natural wing shape variation in *Drosophila *populations is loosely related to individual fitness.

## Background

Phenotypic robustness refers to the invariance of the specified target phenotype given the genetic makeup and environmental conditions. Whereas the presence of naturally occurring phenotypic variation is at the core of evolutionary biology, developmental geneticists have traditionally considered it as a nuisance. Instead, they have relied on the study of single or multiple mutant combinations to reveal the generation of phenotypic patterns (e.g. [1]). A resurgence of interest in the issue of phenotypic robustness has emerged in recent years, partly due to experimental results showing that many knock-out mutations have little effect on phenotype ([2]; although Papp's et al. [3] metabolic network analysis found that the majority of genes that looked dispensable turn out to be such only under laboratory conditions), and that developmental systems show a high degree of stability with respect to perturbations [4,5].

Three major processes are involved in the control of phenotypic variability (the potential or propensity to vary, in the terminology of Wagner and Altenberg [6]): canalization, developmental stability (DS), and plasticity [7]. As first defined by Waddington [8] the term canalization could be understood as a morphogenetic constrain [9], where development appears to be buffered so that slight abnormalities of genotype or slight perturbations in the environment do not lead to the production of abnormal phenotypes. However, evolutionary geneticists define canalization as the tendency of traits to evolve a reduction in variability [4,10]. DS can be defined as the ability of organisms to buffer against the random noise that arises spontaneously as a consequence of stochastic variation in the cellular processes that are involved in the development of morphological structures [11]. Therefore, canalization and DS are subcategories of developmental buffering: the first can be appraised by estimating interindividual variance whereas the most commonly used estimate of DS in bilaterally symmetrical organisms is fluctuating asymmetry (FA); i.e. the intraindividual variation due to random differences between left and right sides. The question of whether or not canalization and DS are different buffering mechanisms has been a constant source of debate. Two recent reviews implicitly [4] or explicitly [10] assume that DS is a special case of canalization, a viewpoint also embraced by several authors (e.g. [12-14]). Thus, by using geometric morphometrics Klingenberg and McIntyre [13] found that the vectors describing inter- and intraindividual variation of landmark position for fly vein traits were highly concordant. On the other hand, Debat et al. [15] came to the opposite conclusion applying the same methods to cranial landmarks in the house mouse – although Klingenberg's et al. [16] work with mouse mandibles found patterns of intra- and interindividual variation that were only partly consistent –. At first glance, the different results may suggest that the mechanisms that affect canalization and DS are related in some developmental contexts but not in others. The problem is, however, that according to the causes of phenotypic variation a distinction between genetic and environmental canalization is necessary [17,18]. Selection for environmental canalization may produce genetic canalization as a by-product [4,10], but this may not always be the case.

The better way to address these contentious issues is to rely on quantitative genetic analyses devised to partition phenotypic variation into genetic and environmental components [19]. Environmental variation can be further partitioned into general () and special (micro) environmental effects (): the first refer to influential factors (e.g. temperature) that are shared by groups of individuals, whereas the latter are residual deviations from the phenotype that would be specified on the basis of genotype and general environmental effects. Such deviations are unique to individuals and are largely unpredictable. The variance associated with special environmental effects can be estimated when experiments are performed on completely inbred lines (i.e., there is no genetic variance). In bilaterally symmetrical organisms it is also feasible to estimate the two sources that contribute to those special environmental effects: among-individual () and within-individual variance (). If the only real cause of asymmetry is variation due to stochasticity in development, then FA can be taken as an estimated of . Therefore, FA is only one source of the phenotypic variation within environments (excluding environmentally induced asymmetry), contrarily to the arguments in Nijhout and Davidovitz [20]. The other source is .

The third process involved in the control of phenotypic variability is plasticity, which can be defined as the ability of an individual to express one phenotype under one set of environmental circumstances and another phenotype under another set. The expressed phenotypes can be discontinuous thus eliciting discrete morphs (i.e., polyphenism), or there can be a continuous range of potential phenotypes (i.e., reaction norm). The reaction norm is thus a property of the genome: genetic canalization and phenotypic plasticity are not mutually exclusive and can combine to form canalized reaction norms [7,17]. Plasticity is thus an alternative to genetic change allowing populations to adapt to changing environmental conditions. To summarize, phenotypic plasticity increases the variance among groups of individuals that produce different phenotypes in different environments, canalization decreases the within-group interindividual variance around the target phenotype by reducing the sensitivity to genetic and environmental conditions, and DS buffers against random perturbations in development (i.e., decreases FA). Because the left and right body sides share the same genome (barring unusual somatic mutation or somatic recombination) and in most organisms also very nearly the same environment, FA provides an intrinsic control for genetic and environmental effects and the important question is to what extent these two sources of variation share underlying regulatory mechanisms.

Within the framework of recently developed geometrically based methods for the statistical analysis of size and shape variation (collectively referred to as geometric morphometrics [21,22]), the wing vein network of *Drosophila *is regarded as an excellent model system to investigate those problems [23,24]. Wing development in *Drosophila *is well understood [25], and the vein pattern is highly conserved across species (e.g. [26]). When flies are reared at low temperatures it is well known that the final wing size increases because of an increase in adult cell size [27]. This plastic response is parallel to what has been commonly observed in laboratory experiments on thermal evolution, where adaptation to lower temperature resulted in increased wing size (a proxy for body size) entirely as a consequence of cell size divergence [28]. However, there is circumstantial evidence suggesting that developmental and evolutionary temperature-related cell size divergence have contrasting effects on wing shape. Thus, Birdsall et al. [29] concluded that wing shape in *Drosophila melanogaster *is quite resistant to developmental temperature. Conversely, in *D. subobscura *there are changes in wing proportions along a latitudinal size cline mediated by cell area [30,31]. These populations exhibit, in addition, prominent latitudinal clines for chromosomal inversion polymorphisms, and there is compelling evidence showing that the inversion clines underlie the latitudinal changes in wing proportions [32,33].

Here we report on the effects of clinal genetic variation (inversion polymorphism) on wing form (size and shape) and bilateral asymmetry using isochromosomal lines of *D. subobscura*. We consider the consequences of inbreeding and temperature on the two components of developmental homeostasis (canalization and DS), and the relationship between them. The remainder of the paper is planned as follows. First, we provide a short account of the inversion polymorphism in *D. subobscura *and the experimental settings. Then, based on the well balanced data set rendered by the experimental design we used the standard least-squares (ANOVA) method to decompose sources of variation for wing size and shape into causal components at the core of further analyses. Furthermore, because the underlying assumption to use FA as a measure of DS is that left – right-side variation has not heritable basis, the genetic and environmental components of bilateral asymmetry were partitioned. As a result, our approach is unusual in studies of DS in providing estimates of the two components of special environmental effects (co-) variance under different genetic backgrounds and general environmental settings. We also present some evidence for the presence of genetic variation in directional asymmetry (DA) but not in FA. Next, we test whether or not the vectors describing variation of landmark position for fly vein traits are concordant, and finally we discuss the main findings in relation with the evolution of buffering mechanisms and the putative adaptive value of natural wing shape variation in *D subobscura*.

## Experimental settings

*D. subobscura *is a particularly inversion-rich species, with up to 38 natural chromosomal arrangements already reported for the largest chromosome O (homologous to arm 3R in *D. melanogaster *[34]) for which a balancer stock is available. In colonizing populations of the New World only six gene arrangements are segregating for that chromosome: O_st_, O_3+4_, O_3+4+2_, O_3+4+7_, O_3+4+8 _and O_5 _(arrangement O_7 _is also present at very low frequency but it is probably the result of a recombination event in the O_st_/O_3+4+7 _heterokaryotype [35]). In native Palearctic populations arrangements O_3+4+2 _and O_3+4+8 _are restricted to the Mediterranean region (the likely area from which the original American colonists derived [36]) and are not involved in latitudinal clines [35]. On the other hand, arrangement O_st _shows a world-wide positive correlation with latitude, while arrangements O_3+4 _and O_3+4+7 _show a contrasting pattern [35]. Therefore, six independent isochromosomal lines for each of these three chromosome arrangements (i.e., , ..., ; *j *= st, 3+4, 3+4+7) were used in the present experiments.

The experimental flies were obtained from 54 crosses, which will be referred to as inbred (isogenic; i.e., ) with 18 crosses in total, or outbred (including both structural homo- and heterokaryotypes) with 36 (18 + 18) crosses in total. The six lines with a given gene arrangement were crossed to produce the three different outbred homokaryotypes (i.e., ). The three kinds of heterokaryotypic flies were similarly obtained but using lines with different gene arrangements (i.e., ). Since all isochromosomal lines were homogeneous for the same genetic background (except for the male sex chromosome), maternal effects were not considered to be critically important. Anyhow, experimental flies were randomly derived form reciprocal crosses for all outbred combinations. Two developmental temperatures were used in the experiment: optimal (18°C) and warm (23°C).

## Results and discussion

### Variation and asymmetry in size

#### a) Basic statistics

Signed left-right () differences of centroid size did not significantly departure from normality in any case (*D*_max _ranging from 0.032 for inbred females at 18°C to 0.073 for inbred males at 23°C; *P *> 0.05). In addition, none of the regressions of centroid size FA on average wing size was statistically significant (ranging from *β *= -0.045 (95% C.I.: -0.091, 0.001) for inbred females at 18°C to *β *= 0.030 (-0.005, 0.064) for inbred females at 23°C), thus suggesting independence between size and size FA.

#### b) Causal components of variation

For each sex two-way mixed ANOVAs were separately performed for inbred and outbred crosses at each experimental temperature (Tables 1, 2, 3, 4). Size variation (CS: centroid size) among individuals comprised the largest part (> 90%) of the variation. The fraction of the total phenotypic variance in wing size associated to genetic differences among karyotypes and/or lines (i.e., ) ranged from 0.235 (inbred males at 18°C) to 0.602 (inbred females at 23°C). (Bear in mind that there is nothing in the ANOVA method of estimation that will prevent a negative variance estimate [37].)

**Table 1:** Asymmetry of overall wing size for females raised at 18°C *Drosophila subobscura *flies raised from inbred (isogenic) and outbred crosses reared at 18°C. Centroid size (CS, estimated in a normalized form [22]) is the dependent variable (values in pixels^2^: 1 mm = 144 pixels). The ANOVAs assess measurement error, directional asymmetry (Sides effect), fluctuating asymmetry (Individuals × Sides interaction effect), and genetic components of the trait () and DA of the trait ((DA)). (CS) and (DA_CS_) provide here unbiased estimates of the among-fly (i.e. ) and within-fly ( or FA) special environmental effects. (⊂ means 'nested in'.)

		Inbred			Outbred		
Source of variation	Variance component	d.f.	Mean Square	Estimated variance	d.f.	Mean Square	Estimated variance

Individuals (I)		107	39.747***	9.6175	215	45.773***	11.2830
Karyotypes (K)	(CS)	2	114.593^n.s.^	0.1389	5	214.204^n.s.^	0.7014
Cross ⊂ K	(CS)	15	94.589***	2.7352	30	113.199***	3.4726
Among flies	(CS)	90	28.944***	6.9166	180	29.857***	7.3040
Sides (S)		1	15.982***		1	18.549***	
I × S	(CS)	107	1.278***	0.5467	215	0.641***	0.2225
Karyotypes (K)	(DA_CS_)	2	0.067^n.s.^	-0.0520	5	0.457^n.s.^	-0.0123
Cross ⊂ K	(DA_CS_)	15	1.938^¶^	0.1239	30	0.899^¶^	0.0492
Within flies	(DA_CS_)	90	1.194***	0.5051	180	0.603***	0.2036
Measurement error	(CS)	216	0.184	0.1841	432	0.196	0.1962

Average CS for left (*L*) and right (*R*) wings: inbred females  = 0.9918 mm,  = 0.9891 ; outbred females  = 1.0022,  = 1.0002.

^n.s. ^*P *> 0.10; ^¶ ^0.10 >*P *> 0.05; *** *P *< 0.001.

**Table 2:** Asymmetry of overall wing size for males raised at 18°C Same as in Table 1.

		Inbred			Outbred		
Source of variation	Variance component	d.f.	Mean Square	Estimated variance	d.f.	Mean Square	Estimated variance

Individuals (I)		107	43.303***	10.4842	215	38.600***	9.4200
Karyotypes (K)	(CS)	2	232.359^¶^	1.1331	5	115.718^n.s.^	0.1908
Cross ⊂ K	(CS)	15	69.186*	1.4332	30	88.246***	2.5026
Among flies	(CS)	90	34.788***	8.3554	180	28.184***	6.8159
Sides (S)		1	1.140^n.s.^		1	22.492***	
I × S	(CS)	107	1.366***	0.5045	215	0.920***	0.3297
Karyotypes (K)	(DA_CS_)	2	0.385^n.s.^	-0.0176	5	1.156^n.s.^	0.0035
Cross ⊂ K	(DA_CS_)	15	1.017^n.s.^	-0.0715	30	1.031^n.s.^	0.0226
Within flies	(DA_CS_)	90	1.446***	0.5444	180	0.895***	0.3172
Measurement error	(CS)	216	0.357	0.3574	432	0.261	0.2609

Average CS for left (*L*) and right (*R*) wings: inbred males  = 0.8942,  = 0.8935; outbred males  = 0.9003,  = 0.8980.

^n.s. ^*P *> 0.10; ^¶ ^0.10 >*P *> 0.05; * *P *< 0.05; *** *P *< 0.001.

**Table 3:** Asymmetry of overall wing size for females raised at 23°C Same as in Table 1 for *Drosophila subobscura *flies reared at 23°C

		Inbred			Outbred		
Source of variation	Variance component	d.f.	Mean Square	Estimated variance	d.f.	Mean Square	Estimated variance

Individuals (I)		107	64.857***	15.8796	215	49.100***	11.9347
Karyotypes (K)	(CS)	2	27.808^n.s.^	-1.8893	5	457.293***	2.6178
Cross ⊂ K	(CS)	15	299.873***	11.3901	30	80.332***	1.9907
Among flies	(CS)	90	26.511***	6.2931	180	32.556***	7.7987
Sides (S)		1	33.413***		1	16.825***	
I × S	(CS)	107	1.339***	0.5446	215	1.361***	0.5916
Karyotypes (K)	(DA_CS_)	2	0.681^n.s.^	-0.0125	5	4.333*	0.0781
Cross ⊂ K	(DA_CS_)	15	1.132^n.s.^	-0.0427	30	1.520^n.s.^	0.0447
Within flies	(DA_CS_)	90	1.388***	0.5692	180	1.252***	0.5371
Measurement error	(CS)	216	0.250	0.2496	432	0.178	0.1778

Average CS for left (*L*) and right (*R*) wings: inbred females  = 0.8999 mm,  = 0.8960; outbred females  = 0.9203,  = 0.9184.

^n.s. ^*P *> 0.10; * *P *< 0.05; *** *P *< 0.001.

**Table 4:** Asymmetry of overall wing size for males raised at 23°C Same as in Table 1 for *Drosophila subobscura *flies reared at 23°C

		Inbred			Outbred		
Source of variation	Variance component	d.f	Mean Square	Estimated variance	d.f.	Mean Square	Estimated variance

Individuals (I)		107	44.690***	10.9045	215	28.772***	6.8138
Karyotypes (K)	(CS)	2	41.926^n.s.^	-0.6021	5	112.284^n.s.^	0.3480
Cross ⊂ K	(CS)	15	128.628***	4.0778	30	62.165***	1.7199
Among flies	(CS)	90	30.762***	7.4224	180	20.887***	4.8425
Sides (S)		1	36.691***		1	13.586**	
I × S	(CS)	107	1.072***	0.3553	215	1.517***	0.6465
Karyotypes (K)	(CS)	2	2.596^n.s.^	0.0366	5	0.862^n.s.^	-0.0110
Cross ⊂ K	(DA_CS_)	15	1.277^n.s.^	0.0454	30	1.259^n.s.^	-0.0532
Within flies	(DA_CS_)	90	1.004***	0.3213	180	1.578***	0.6771
Measurement error	(CS)	216	0.361	0.3615	432	0.224	0.2240

Average CS for left (*L*) and right (*R*) wings: inbred males  = 0.8112,  = 0.8072 ; outbred males  = 0.8277,  = 0.8260.

^n.s. ^*P *> 0.10; ** *P *< 0.01; *** *P *< 0.001.

**Figure 1:**
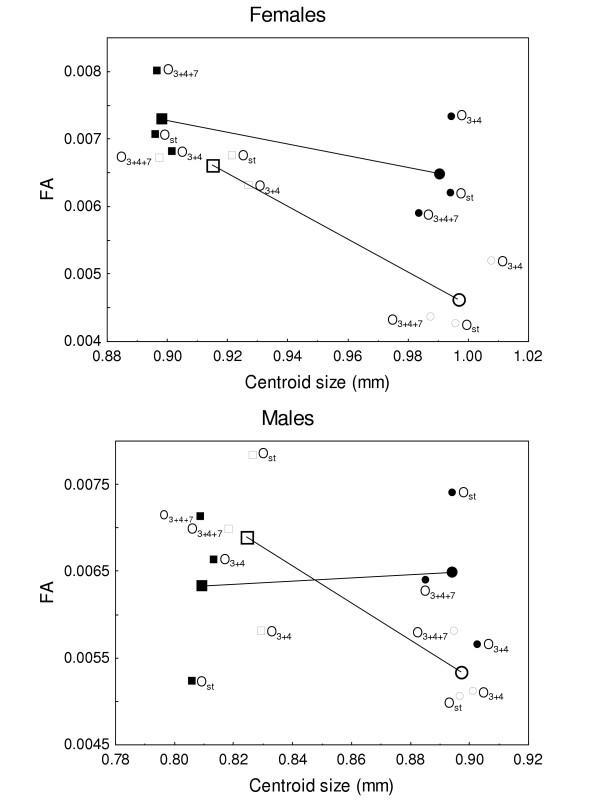
**Inbreeding and temperature effects on size **Homokaryotipic averages for centroid size and centroid size FA (index FA1 in [39]) in inbred (black symbols) and outbred (open symbols) crosses. Small symbols give the average values for each of the three different homokaryotypes to appreciate the dispersion from the corresponding grand average (large symbols connected by lines). Squares give the values at 23°C and circles at 18°C.

No significant size differences were generally detected among karyotypes for average CS, although O_3+4 _flies were always the biggest within inbred lines (Fig. 1). On the other hand, in outbred crosses heterokaryotypes were bigger than homokaryotypes (females: 18°C *F*_(1,195) _= 9.78, *P *= 0.002; 23°C *F*_(1,195) _= 9.19, *P *= 0.003; males: 18°C *F*_(1,195) _= 1.84, *P *= 0.176; 23°C *F*_(1,195) _= 4.23, *P *= 0.041), but interactions of dominance effects were observed in all samples with discernible heterosis in O_st_/O_3+4 _lines when compared to their homokaryotypic counterparts.

In concert with some independent preliminary results using a set of O_st _isochromosomal lines [38] a quite remarkable finding here was that left wings were consistently bigger than the right ones, thus causing a generally highly significant DA (i.e., "sides" effect in Tables 1, 2, 3, 4) of overall wing size even though DA was fairly subtle (see bottom statistics in Tables 1, 2, 3, 4). Each *Drosophila *wing vein has dorsal and ventral components that come together after the apposition of the dorsal and ventral surfaces, but each vein protrudes only in one wing surface ("corrugation") [25]. When wings were mounted no attempt was made to standardize the surface position: in females 394 (60.8%) left and 387 (59.7%) right wings were mounted on the slides with the dorsal side up (*χ*^2 ^= 0.16, 1 df, *P *= 0.691); in males the corresponding figures were 383 (59.1%) and 401 (61.9%), respectively (*χ*^2 ^= 1.05, 1 df, *P *= 0.306). Potential biasing effects when measuring wings; namely, dorsal or ventral Bitmap images or possible differences between left and right wings when Bitmap images are captured from the top or bottom of the microscope slide, were checked from a subset of 75 females and 75 males. An additional set of two images for each wing were taken in the same session from the top and bottom of the slide and digitized once. The centroid size differences between the averages of both measurements was apparently random with respect to digitizing procedure and always lower than 0.07%, whereas left wings were 0.26% bigger than the right ones in females and 0.34% in males. We are, therefore, quite confident that the fairly subtle DA for wing CS is not an experimental artifact but a real phenomenon.

In addition to DA, there was subtle but significant FA in all crosses (i.e., "individuals × sides" interaction effect in Tables 1, 2, 3, 4) together with a small amount of genetic variation for DA in some of them. This last finding could hardly be attributable to a type I error because similar results had been previously obtained [[38]; see below]. Conversely, two-level nested ANOVAs to test for genetic components of overall size FA (using index FA1 in Palmer [39]) failed to show any statistically significant effects whatsoever (variance components ranging from -0.0047 to 0.0071 for karyotypes, and from -0.0343 to 0.0406 for crosses within karyotypes; values in pixels^2^).

#### c) Consanguinity and temperature effects

Inbreeding and environmental effects were simultaneously analyzed by contrasting isogenic *vs*. outbred homokaryotypic flies reared at both experimental temperatures (Fig. 1). Flies were obviously bigger when raised at the lowest temperature, and three-way factorial ANOVAs performed separately for each sex using CS (as log_*e *_(pixels), but results were qualitatively identical without a log-transformation) as the dependent variable, with karyotype, temperature and inbreeding as fixed effects, and crosses nested within karyotypes, clearly indicated inbreeding depression together with temperature by inbreeding interaction (i.e., inbreeding was most noticeable at the sub-optimal temperature of 23°C), but no karyotype by temperature interaction was detected. These results confirm that wing size is not a purely additive trait in *D. subobscura*, in agreement with the previous observation that heterokaryotypes were bigger than homokaryotypes in outbred crosses (see also [40]).

Both inbreeding and (sub-optimal) temperature effects were also apparent in females when overall size FA (index FA1) was used as the dependent variable in three-way factorial ANOVAs, with no differences among karyotypes. On the other hand, no statistically significant effects were detected for males, basically because inbred crosses performed approximately equal at both temperatures (Fig. 1). However, overall asymmetry augmented in inbred crosses because DA largely increased (mainly in males) at the highest temperature ("temperature × inbreeding" interaction: *F*_(1,856) _= 9.46, *P *= 0.002).

It is worth mentioning here that in outbred crosses overall size FA was about the same for homokaryotypes and heterokaryotypes: the only significant effect was again an increase in FA at the sub-optimal temperature (more than two-fold; c.f. (DA_CS_) values in Tables 1, 2, 3, 4). Finally, inbreeding appears to have affected among-fly variation only in males as suggested by the consistently lower (CS) estimates in outbred crosses within rearing temperature.

In conclusion, overall size DS was positively correlated with levels of heterozygosity (i.e., inbred vs. outbred homokaryotypes) and development at the optimal temperature of 18°C. However, no positive association was found between DS and chromosomal heterozygosity in outbred crosses.

### Variation and asymmetry in shape

#### a) Sources of variation

Two-way MANOVA analyses to quantify inter- and intra-individual variation in wing shape are shown in Tables 5, 6, 7, 8. For the present study of 13 landmarks, with 2 coordinates each, the shape dimension is 22. Sums of squares and cross-products (SSCP) matrices are therefore not full-ranked, and we retained 22 PC (principal components [41]) scores for outbred crosses and only 15 PC scores – which accounted for more than 98% of the total shape variance – for inbred crosses to be capable of testing for genetic components. The degrees of freedom in Tables 5, 6, 7, 8 (columns "df 1") are simply the corresponding degrees of freedom in the ANOVAs for centroid size (Tables 1, 2, 3, 4) times the number of PC scores retained in each sample. Likewise, the overall covariation in wing shape ("individuals" effect) was decomposed into causal components (karyotypes, crosses in karyotypes, and among flies); and the overall covariation in wing shape FA ("individuals × sides" interaction effect) was decomposed into causal components attributable to wing shape DA (karyotypes, crosses in karyotypes, and within flies).

**Table 5:** Asymmetry of overall wing shape for females raised at 18°C Flies raised from inbred (isogenic) and outbred crosses of *Drosophila subobscura *reared at 18°C. For the inbred crosses 15 PC scores were retained for analyses (proportion of total shape variance accounted is given in parenthesis). For the outbred crosses 22 PC scores were retained. (⊂ means 'nested in'.)

	Inbred (98.6%)				Outbred			
Source of variation	Wilks' lambda	df 1	df 2	*P*	Wilks' lambda	df 1	df 2	*P*

								
Individuals (I)	1.13 × 10^-11^	1605	1464	<0.001	5.14 × 10^-15^	4730	4442	<0.001
Karyotypes (K)	0.002	30	2	0.505	7.44 × 10^-5^	110	48	<0.001
Cross ⊂ K	1.51 × 10^-4^	225	843	<0.001	3.68 × 10^-4^	660	2932	<0.001
Among flies	2.23 × 10^-8^	1350	1457	<0.001	7.55 × 10^-12^	3960	4430	<0.001
Sides (S)	0.597	15	93	<0.001	0.563	22	194	<0.001
I × S	1.58 × 10^-9^	1605	3083	<0.001	6.46 × 10^-11^	4730	9192	<0.001
Karyotypes (K)	0.003	30	2	0.546	0.002	110	48	0.301
Cross ⊂ K	0.074	225	843	0.362	0.018	660	2932	0.023
Within flies	9.69 × 10^-9^	1350	3070	<0.001	7.91 × 10^-10^	3960	9169	<0.001

**Table 6:** Asymmetry of overall wing shape for males raised at 18°C Same as in Table 5.

	Inbred (98.5%)				Outbred			
Source of variation	Wilks' lambda	df 1	df 2	*P*	Wilks' lambda	df 1	df 2	*P*

								
Individuals (I)	7.18 × 10^-12^	1605	1464	<0.001	3.51 × 10^-14^	4730	4442	<0.001
Karyotypes (K)	0.004	30	2	0.633	2.49 × 10^-4^	110	48	0.006
Cross ⊂ K	1.61 × 10^-4^	225	843	<0.001	2.98 × 10^-4^	660	2932	<0.001
Among flies	1.47 × 10^-8^	1350	1457	<0.001	3.62 × 10^-11^	3960	4430	<0.001
Sides (S)	0.658	15	93	<0.001	0.569	22	194	<0.001
I × S	5.58 × 10^-8^	1605	3083	<0.001	1.28 × 10^-10^	4730	9192	<0.001
Karyotypes (K)	0.004	30	2	0.605	0.003	110	48	0.449
Cross ⊂ K	0.068	225	843	0.236	0.019	660	2932	0.036
Within flies	3.05 × 10^-7^	1350	3070	<0.001	1.73 × 10^-9^	3960	9169	<0.001

**Table 7:** Asymmetry of overall wing shape for females raised at 23°C Same as in Table 5 for *Drosophila subobscura *flies reared at 23°C.

	Inbred (98.3%)				Outbred			
Source of variation	Wilks' lambda	df 1	df 2	*P*	Wilks' lambda	df 1	df 2	*P*

								
Individuals (I)	1.07 × 10^-12^	1605	1464	<0.001	1.08 × 10^-13^	4730	4442	<0.001
Karyotypes (K)	2.18 × 10^-4^	30	2	0.200	0.001	110	48	0.146
Cross ⊂ K	3.31 × 10^-4^	225	843	<0.001	1.81 × 10^-4^	660	2932	<0.001
Among flies	2.32 × 10^-9^	1350	1457	<0.001	1.54 × 10^-10^	3960	4430	<0.001
Sides (S)	0.450	15	93	<0.001	0.585	22	194	<0.001
I × S	2.57 × 10^-9^	1605	3083	<0.001	3.21 × 10^-13^	4730	9192	<0.001
Karyotypes (K)	0.007	30	2	0.725	0.006	110	48	0.842
Cross ⊂ K	0.055	225	843	0.062	0.034	660	2932	0.889
Within flies	1.95 × 10^-8^	1350	3070	<0.001	3.54 × 10^-12^	3960	9169	<0.001

**Table 8:** Asymmetry of overall wing shape for males raised at 23°C Same as in Table 5 for *Drosophila subobscura *flies reared at 23°C.

	Inbred (98.3%)				Outbred			
Source of variation	Wilks' lambda	df 1	df 2	*P*	Wilks' lambda	df 1	df 2	*P*

								
Individuals (I)	5.39 × 10^-12^	1605	1464	<0.001	6.41 × 10^-14^	4730	4442	<0.001
Karyotypes (K)	8.81 × 10^-4^	30	2	0.364	1.18 × 10^-4^	110	48	<0.001
Cross ⊂ K	2.75 × 10^-4^	225	843	<0.001	1.96 × 10^-4^	660	2932	<0.001
Among flies	8.92 × 10^-9^	1350	1457	<0.001	7.94 × 10^-11^	3960	4430	<0.001
Sides (S)	0.642	15	93	<0.001	0.540	22	194	<0.001
I × S	8.58 × 10^-9^	1605	3083	<0.001	9.84 × 10^-12^	4730	9192	<0.001
Karyotypes (K)	5.13 × 10^-5^	30	2	0.102	6.40 × 10^-4^	110	48	0.052
Cross ⊂ K	0.060	225	843	0.111	0.024	660	2932	0.250
Within flies	5.79 × 10^-8^	1350	3070	<0.001	1.26 × 10^-10^	3960	9169	<0.001

Similarly to what had been found for CS, differences between left and right wings were also highly significant ("sides" effect), thus indicating that DA was present for overall wing shape. This finding is contrary to our previous claim from a subset of O_st _isochromosomal lines, where DA for some landmarks (e.g. those defining the position of the anterior crossvein) but not for overall wing shape was detected [38]. After plotting the Procrustes grand mean shapes of both wings it also became apparent here that the location of the anterior crossvein was indeed slightly more distal in the right wings. Furthermore, the individuals × sides interaction effects were highly significant in all cases and, hence, wing shape FA greatly exceeded measurement error.

#### b) Causal components of variation

As has been forcefully stressed [42] shape is an inherently multidimensional concept and cannot be easily reduced to a scalar index without severe loss of information. Therefore, for a quantitative genetic analysis of shape data a multivariate approach is required [43]. For overall wing shape, genetic differences among karyotypes were mostly detected for outbred crosses (Tables 5, 6, 7, 8), and we have estimated the covariance matrices **P **= **K **+ **C **+ **E **as a simple multivariate extension of the two-level nested ANOVAs, where **P **is the phenotypic covariance matrix and **K**, **C**, and **E **are, respectively, the covariance matrices for karyotypes, crosses within karyotypes, and the residuals.

**Figure 2:**
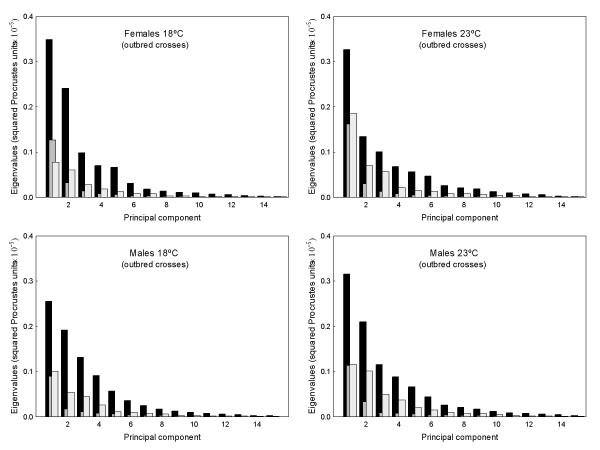
**Eigenvalues of causal covariance matrices for wing shape **First 15 eigenvalues of the phenotypic (black bars), karyotype (hatched) and crosses (open) covariance matrices from outbred crosses.

Fig. 2 shows the amount of variation associated with the different dimensions in shape space. Much of the variation was concentrated in the first few PCs, but the **K **matrices showed the clearest trend to quickly decrease after the first PC. Permutation tests indicated that matrix correlations (MCs) between **K **and **C **matrices were generally higher at 18°C (females MC = 0.258, *P *= 0.1908 ; males MC = 0.305, *P *= 0.1963) than at 23°C (females MC = 0.157, *P *= 0.3356 ; males MC = 0.250, *P *= 0.2665), but none of the MCs was statistically significant. On the other hand, VCV matrices were correlated across rearing temperatures (females: MC **K **= 0.716, *P *= 0.0163; MC **C **= 0.818, *P *= 0.0001; males: MC **K **= 0.706, *P *= 0.0160 ; MC **C **= 0.587, *P *= 0.0399 ; this last correlation was no longer significant after the Bonferroni procedure [44]). A close inspection to Fig. 2 reveals an increase in the genetic components of overall wing shape at 23°C, which agrees with our preliminary findings [38]. Thus, the ratio between the total variance of genetic (**G **= **K **+ **C**) covariance matrix onto the total variance of the phenotypic covariance matrix was lower at 18°C in both sexes (females: 0.1312 *vs*. 0.5450; males: 0.2365 *vs*. 0.2522). A caveat: these ratios cannot be interpreted as estimates of shape heritability [43].

MANOVA results in Tables 5, 6, 7, 8 also point to the presence of genetic variation for overall shape DA, mainly at 18°C (i.e., the "crosses in karyotypes" component from the decomposition of the I × S interaction effect). As far as we are aware, these are the first experiments that found detectable genetic variation in DA for wing traits. The uncovering of DA (i.e., "side" effect) for fly wings is quite general when quantitative analyses of form are carried out using the powerful methods of geometric morphometrics to reveal even small morphological variation that otherwise would remain hidden with less effective techniques [13,45]. This has raised concerns against the conventional wisdom that left and right are not distinguished in *Drosophila *development [46] because it provides compelling evidence that DA in fly wings may signal the presence of genetic variation in a phylogenetic conserved left-right developmental axis (i.e., an imaginary plane between the two lateral sides of the body), as discussed by Klingenberg et al. [45]. Actually, modern treatises in developmental biology (e.g. [9]) distinguish the left-right axis besides the customary anterior-posterior and dorsal-ventral axes, and several asymmetrically expressed genes (e.g. *sonic hedgehog*) have recently been discovered. In *Drosophila*, Ligoxygakis et al. [47] were the first (and to our knowledge the only ones) who showed a developmental mechanism for the developmental asymmetry. It seems, therefore, that the detection of genetic variation for DA in this genus appears to be basically a methodological problem, including statistical power and the environmental conditions where the experiments are performed. The mechanisms that constitute the genetic basis of morphological asymmetry in *Drosophila *obviously require further study.

#### c) Genetic components of wing shape FA

Following [13] a multivariate equivalent of FA1 (i.e., the "unsigned" left-right differences) was defined by changing the signs of all coordinate differences (from left-right to right-left) whenever the inner product (also referred to as the dot product) of a left-right difference vector with the vector of mean left-right difference was negative. For the univariate case (CS) this procedure would render here the absolute () differences, but notice that for the multivariate case it is not equivalent to calculate the absolute () differences of all Procrustes coordinates.

MANOVA analyses of these "unsigned" shape asymmetries in outbred crosses did not detect any genetic variation for shape FA at 18°C or 23°C (Tables 9, 10). However, the approach used to define the multivariate equivalent of FA1 might be influenced by the arbitrary choice of the plane (i.e., the mean left-right differences) to subdivide the shape space into "positive" and "negative" halves (Christian P Klingenberg, pers. comm. 2004). A modified Procrustes shape distance for non-isotropic variation (i.e., landmarks usually differ in their amounts of variation) has been recently developed by Klingenberg and Monteiro [48], and can be used here as a scalar measure of the amount of shape asymmetry because FA is random in origin (i.e., only the magnitude and not the direction may usually be the interesting component of FA shape variation). When this scalar was used in our data set the same conclusion was obtained; namely, there was no detectable genetic variation for wing shape FA in any case (results not shown).

**Table 9:** MANOVAs for female wing shape fluctuating asymmetry A multivariate equivalent of FA1 (i.e., the "unsigned" left-right differences) was defined as explained in the text. Flies raised from outbred crosses of *Drosophila subobscura *(⊂ means 'nested in').

	18°C				23°C			
Source of variation	Wilks' lambda	df 1	df 2	*P*	Wilks' lambda	df 1	df 2	*P*

Karyotypes (K)	0.008	110	48	0.908	0.007	110	48	0.856
Cross ⊂ K	0.022	660	2932	0.169	0.029	660	2932	0.604

**Table 10:** MANOVAs for male wing shape fluctuating asymmetry Same as in Table 9.

	18°C				23°C			
Source of variation	Wilks' lambda	df 1	df 2	*P*	Wilks' lambda	df 1	df 2	*P*

Karyotypes (K)	0.004	110	48	0.627	0.009	110	48	0.938
Cross ⊂ K	0.024	660	2932	0.243	0.042	660	2932	0.988

#### d) Consanguinity and temperature effects on wing shape

To investigate allometric and nonallometric temperature effects on overall wing shape we performed a multivariate analyses of covariance (MANCOVA) of the Procrustes coordinates (after averaging both sides and the two replicated measurements per side) considering temperature and inbreeding (i.e., isogenic *vs*. outbred homokaryotypic flies) as the categorical predictors and CS (as log_*e *_(pixels)) as the covariate. Temperature effects were only significant in males, but inbreeding and temperature × inbreeding interaction effects were highly significant in both sexes (results not shown), which suggests a strong effect of the categorical predictors on the nonallometric component of shape. Size effects were also found to be significant (females: Wilks' *λ *= 0.881, *F*_(22,405) _= 2.496, *P *< 0.001; males: Wilks' *λ *= 0.915, *F*_(22,405) _= 1.715, *P *= 0.024), but the allometric effect on shape remained relatively consistent at both temperatures in females (size × temperature interaction: Wilks' *λ *= 0.930, *F*_(22,405) _= 1.395, *P *= 0.111) but not in males (Wilks' *λ *= 0.853, *F*_(22,405) _= 3.165, *P *<0.001). The association between size and temperature (Fig. 1), measured by the variance inflaction factor (*VIF *< 5; [49]), was found to be lower than the suggested guideline for serious collinearity (i.e. *VIF *≥ 10), which indicates that the effects of temperature and size on wing shape could be effectively separated.

The conclusion is, therefore, that *Drosophila *wing shape does not seem to be as resistant to environmental temperature as previously claimed from the analysis of 12 highly inbred *D. melanogaster *lines [29].

Inbreeding effects (isogenic *vs*. outbred homokaryotypic flies) on wing shape FA were tested from the ratio between the traces of the corresponding "individual × side" VCV matrices. Notice that the traces of these interaction matrices are equal to the respective mean squares of the Procrustes ANOVA as implemented by Klingenberg and McIntyre [13], and are simply the sum of  (index FA4 in [39]) for each *x *and *y *coordinates of the corresponding aligned configurations divided by the shape dimension. We performed 10,000 randomization runs for each test. Inbreeding effects were detected at 18°C but only in females (18°C: female *F *= 1.694, *P *= 0.0003 ; male *F *= 0.963, *P *= 0.6037 ; 23°C: female *F *= 0.834, *P *= 0.9231; male *F *= 0.984, *P *= 0.5541).

### Patterns of wing shape variation

#### a) Fluctuating asymmetry

**Figure 3:**
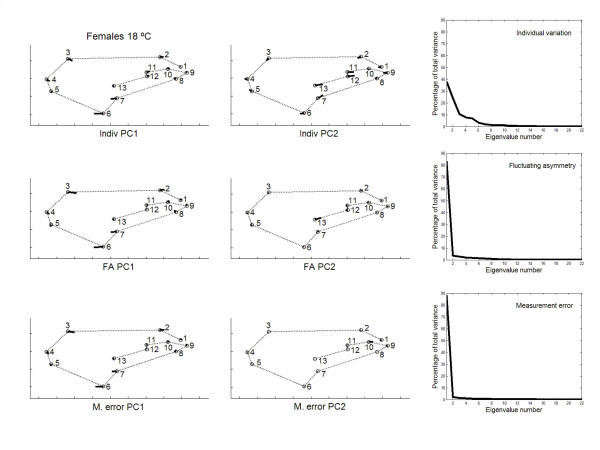
**Vectors of the landmarks displacements **First two axes of wing shape variation for each effect in the two-way mixed MANOVA (individuals, individuals × sides interaction, and measurement error) for females from outbred crosses reared at 18°C. Also plotted are the percentages of total wing shape variation explained by the principal components for the corresponding covariance matrices.

**Figure 4:**
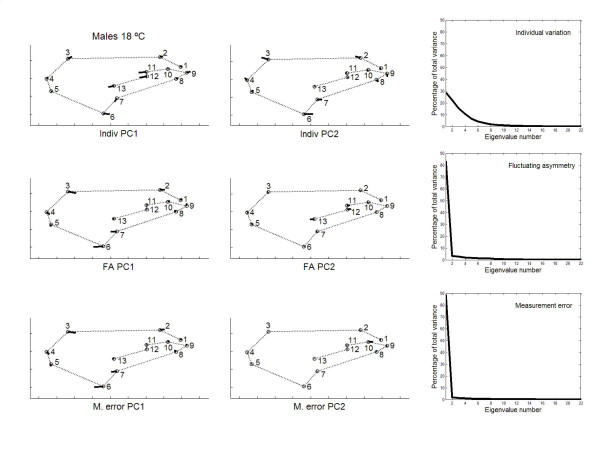
**Vectors of the landmarks displacements **Same as Fig. 3 for males from outbred crosses reared at 18°C.

**Figure 5:**
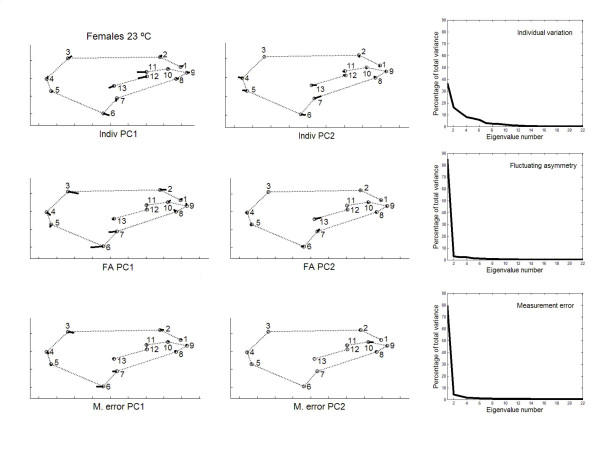
**Vectors of the landmarks displacements **Same as Fig. 3 for females from outbred crosses reared at 23°C.

**Figure 6:**
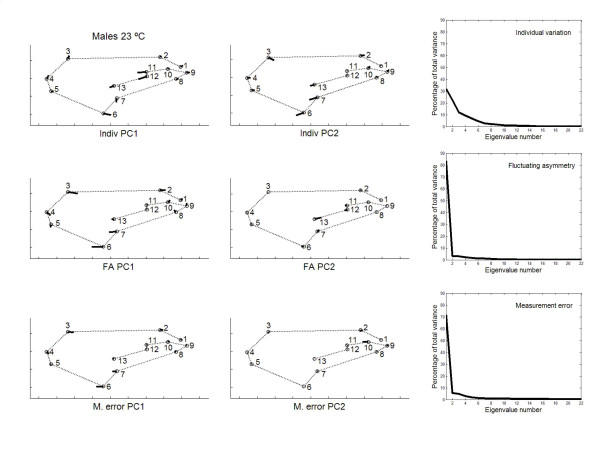
**Vectors of the landmarks displacements **Same as Fig. 3 for males from outbred crosses reared at 23°C.

Principal component analyses were only implemented for the outbred crosses since they are more representative of the natural situation. The percentages of total shape variation, together with the features of variation associated with the dominant PCs, are graphically plotted in Figs. 3, 4, 5, 6. For the individual variation several PCs accounted for relatively large amounts of variability. On the contrary, for FA and measurement error PC1 explained almost all total variance (>80%). For all levels in the analysis (i.e. individuals, FA and measurement error) the dominant PCs were connected to the relatively large variability of landmarks 3, 6, 7 and, to a lesser extent, landmark 2. However, the disproportionate amount of variation associated with these landmarks did not spread to all sources of causal variation because their coefficients were relatively small for the PC1 of karyotype variation (which explained ~60% of the total variance; see below). Furthermore, for the individual variation the first two PCs were also linked to the shift of the anterior (landmarks 11 and 12) and posterior (landmarks 7 and 13) crossveins along the adjoining longitudinal veins.

Permutation tests indicated that VCV matrices were mostly correlated for FA and measurement error effects within samples (MCs > 0.95, *P *< 0.01; Table 11). The individual VCV matrix was significantly correlated with the FA and measurement error matrices only for females at 18°C. Between temperatures the VCV matrices were highly correlated for FA and measurement error (results not shown), but loosely correlated for the individual variation (females MC = 0.668, *P *= 0.0355 ; males MC = 0.494, *P *= 0.1066 ; statistical significance vanishes after the Bonferroni procedure).

**Table 11:** Correlations between VCV matrices of landmarks displacements within groups Results of the permutation tests used for the analyses within sexes and temperatures.

Group	Effects	Correlation	*P *(permutation)	*P *(Bonferroni)
Females 18°C	Individual / FA	0.7699	0.0001	**
	Karyotype / FA	-0.1691	0.7583	n.s.
	Cross / FA	0.5773	0.0871	n.s.
	Between-fly / FA	0.7517	0.0001	**
	Individual / error	0.7550	0.0001	**
	FA / error	0.9953	0.0001	**
				
Males 18°C	Individual / FA	-0.3998	0.8694	n.s.
	Karyotype / FA	0.0067	0.4393	n.s.
	Cross / FA	-0.0706	0.6202	n.s.
	Between-fly / FA	0.2060	0.3296	n.s.
	Individual / error	-0.4280	0.9436	n.s.
	FA / error	0.9964	0.0001	**
				
Females 23°C	Individual / FA	0.1233	0.2881	n.s.
	Karyotype / FA	-0.0151	0.4771	n.s.
	Cross / FA	0.6516	0.0264	n.s.
	Between-fly / FA	0.5764	0.0744	n.s.
	Individual / error	0.1093	0.3141	n.s.
	FA / error	0.9959	0.0001	**
				
Males 23°C	Individual / FA	0.5278	0.0523	n.s.
	Karyotype / FA	-0.1469	0.7545	n.s.
	Cross / FA	0.2752	0.1817	n.s.
	Between-fly / FA	0.4033	0.1241	n.s.
	Individual / error	0.5165	0.0519	n.s.
	FA / error	0.9922	0.0001	**

n.s. = *P *> 0.05; ** = *P *< 0.01.

The angles between the PC1s for FA and measurement error were very much alike (ranging from angle *α *= 2.1° to *α *= 3.4° ; recall that the 0.1% quantile of the resulting distribution between pairs of random vectors in 22-dimensional space was 50.3°), which reflects the similarity due to landmarks 3, 6 and 7. However, the first three PCs for interindividual variation were generally distinct to those of FA: the only clear correspondences were between the PC1s for females at 18°C (*α *= 21.5°), and the PC2 of interindividual variation with the PC1 of FA for males at 18°C (*α *= 11.8°). (The correspondences were qualitatively the same for interindividual variation and measurement error; results not shown.) Overall, these results seem to suggest that canalization and DS do not generally share the same underlying regulatory mechanisms (but see below).

A potentially important problem with the foregoing approach to compare the patterns of intra- and interindividual variation is to rely on the interaction VCV matrix as the source of variation due to FA. As has been previously argued the uncovering of DA is almost ubiquitous for shape data when using the methods of geometry morphometrics, and there was evidence here for statistically significant genetic variation of overall shape DA at 18°C (Tables 5, 6). Therefore, the VCV matrix from the "individuals × sides" interaction effect gives a biased estimate of developmental stability and cannot be taken as the covariance matrix for FA. In other words, this VCV matrix also includes all causal components due to genetic variation for DA, and the corresponding unbiased VCV matrix for FA is that for the within-fly component of the interaction effect (i.e., after removing the genetic variation for DA [50,51]). In any case, all results were qualitatively similar and, hence, the conclusion that canalization and DS seem to be different mechanisms remains unchanged. However, it is difficult to appraise how this potential problem could have affected the previously published conclusions when comparing interindividual variation and "FA" in fly wings and mouse skulls (see Background section).

Between rearing temperatures the congruence of PC1 eigenvectors was also very high for FA (females *α *= 4.0°; males *α *= 3.5°) and measurement error (females *α *= 3.1°; males *α *= 4.1°). For the interindividual variation the correlations between PC1s were significant only in males (females *α *= 74.3°; males *α *= 19.3°); however, the PC1 vector describing the joint interindividual variation of landmark position in females at 18°C matched the PC2 of the interindividual covariance matrix at 23°C (*α *= 49.6°; recall that the direction of PCs is arbitrary and all the movements in Figs. 3, 4, 5, 6 can be simultaneously reversed by 180°) and *vice versa *(i.e., PC1 at 23°C *vs *PC2 at 18°C: *α *= 26.4°).

#### b) Causal components

**Figure 7:**
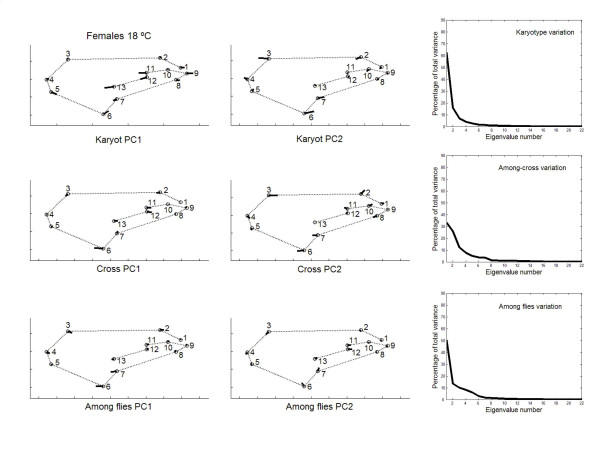
**Vectors of the landmarks displacements **First two axes of wing shape variation in the two-level nested MANOVA (karyotypes, crosses nested in karyotypes, and within crosses) for each causal component effect pertaining to the inter-individual variation in females from outbred crosses reared at 18°C. Also plotted are the percentages of total wing shape variation explained by the principal components for the corresponding covariance matrices.

**Figure 8:**
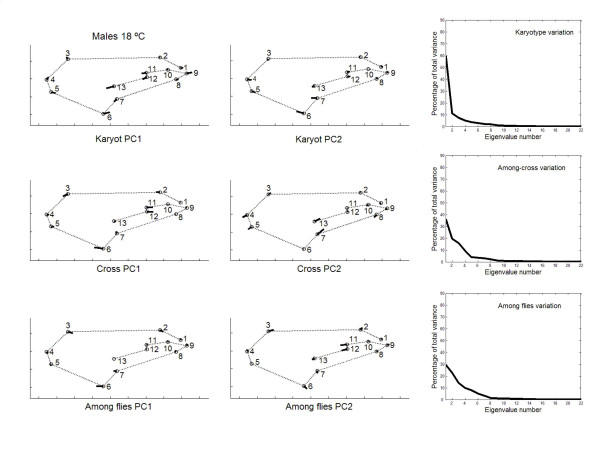
**Vectors of the landmarks displacements **Same as Fig. 7 for males from outbred crosses reared at 18°C.

**Figure 9:**
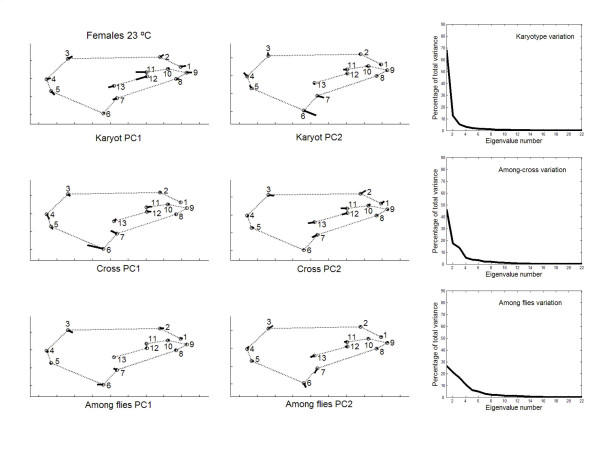
**Vectors of the landmarks displacements **Same as Fig. 7 for females from outbred crosses reared at 23°C.

**Figure 10:**
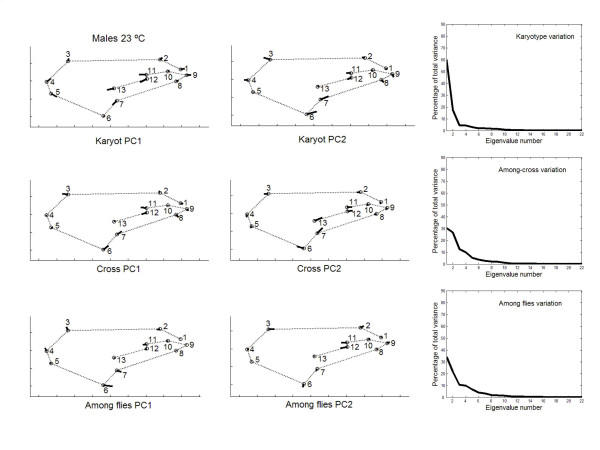
**Vectors of the landmarks displacements **Same as Fig. 7 for males from outbred crosses reared at 23°C.

**Figure 12:**
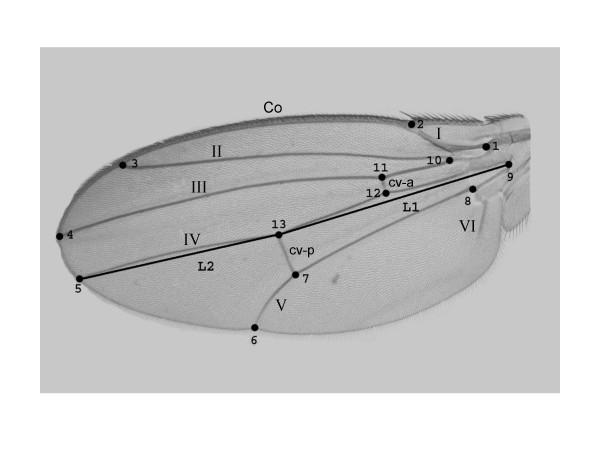
**Left wing of *Drosophila subobscura ***The image shows the thirteen landmarks (1 – 13) used in this work. I – VI longitudinal veins; cv-a and cv-p anterior and posterior crossveins; Co costal or marginal veins; L1 and L2 lengths of the proximal (Euclidian distance between landmarks 9 and 13) and distal (Euclidian distance between landmarks 13 and 5) segments of longitudinal vein IV, respectively. Wing shape index  has been previously used to study shape clines in this species [30].

Besides the interindividual variation in the two-way MANOVAs (which comprises genetic plus environmental covariances due to special environmental effects) it is important here to asses the patterns of joint displacements of landmarks for each of the causal components of wing shape variation (Figs. 7, 8, 9, 10). For karyotype variation PC1 accounted for ~60% of the total variance and was linked to a great extent with equivalent movements of those landmarks defining the location of the crossveins, which shifted in the same direction. Landmarks 4 and 5 tended to move away each other, stretching the wing margin between longitudinal veins III and IV. Landmark 9 budged in the opposite direction to crossveins shifts, thus shaping the relationship between L1 to the total length of longitudinal vein IV (i.e. shape index L1 WL ; Fig. 12).

**Figure 11:**
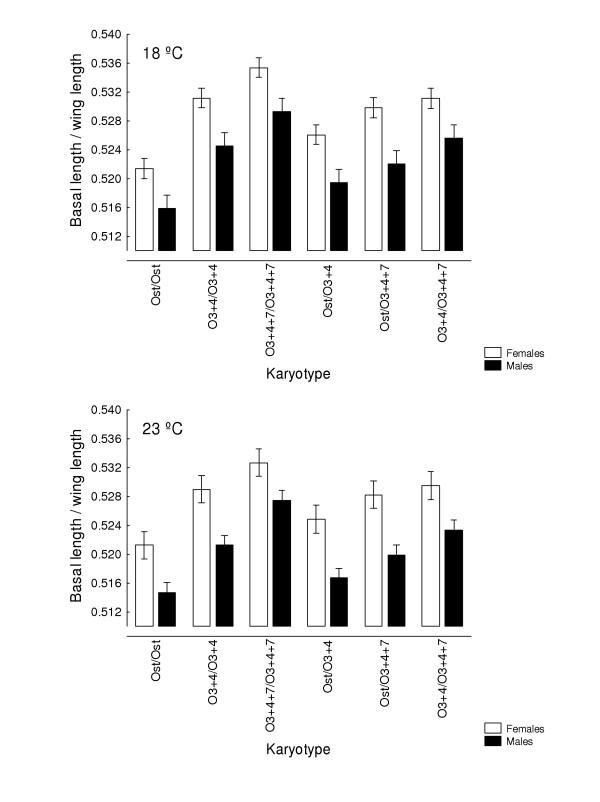
**Wing shape index **Averages of the relative length (with 95% confidence intervals) of the basal portion of longitudinal vein IV (L1) to the total wing length (WL = L1 + L2) versus karyotype for outbred crosses at the two rearing temperatures. A two-way factorial ANOVA using the shape index as , with karyotype and temperature as fixed effects, and crosses nested within karyotypes, detected statistically significant differences for the main effects (karyotype: female *F*_5,30 _= 12.625, *P *< 0.001; male *F*_5,30 _= 9.785, *P *< 0.001. Temperature: female *F*_1,390 _= 30.219, *P *< 0.001; male *F*_1,390 _= 61.835, *P *< 0.001) but no karyotype × temperature interaction (females: *F*_5,390 _= 1.570, *P *= 0.168; males: *F *_5,390 _= 1.111, *P *= 0.354).

A relative shortening of the basal length of longitudinal vein IV relative to the total wing length with an increasing dose of standard gene arrangements in all five major chromosomes of *D. subobscura *had been previously identified in an outbred stock [32,33]. A similar pattern regarding O_st _dose is also clear here when considering the six karyotypes (Fig. 11), but rearing temperature quantitatively modified the shape index (L1/WL was lower at the highest temperature). However, there was no statistically significant karyotype × temperature interaction. The wing shape index appears to be a purely additive trait since heterokaryotypes were always intermediate to their corresponding homokaryotypes (Fig. 11). Actually, none of 12 within- group (i.e., sex and temperature) possible contrasts comparing all three heterokaryotypes with the average of the corresponding homokaryotypes was statistically significant (the mean square for "crosses" was used as the error term; see legend in Fig. 11).

PC2 for karyotypes was also connected to the variability of landmarks 3, 6 and 7. For the crosses component, several PCs explained relatively large amounts of variation, and shifts of crossveins now seem to be independent of each other at 18°C but not at 23°C. Finally, for the within-fly variation several PCs accounted for relatively large amounts of variability. PC1s were again connected to the variability of landmarks 3, 6 and 7; and PC2s to shifts in the anterior crossvein.

The large amount of variation of the anterior and posterior crossveins for karyotypes and crosses can be interpreted in terms of developmental processes. The crossveins are determined after the longitudinal veins, and mutations that eliminate crossveins (e.g. *crossveinless*) do not affect the longitudinal veins; however, some mutants that affect the longitudinal veins also influence the crossveins (e.g. the *vn *group in [1]). Intra- and interespecific studies in several *Drosophila *species have found displacements of one or both crossveins along their longitudinal veins, and such shifts also occur in a number of mutants (see [23]). However, these shifts do not occur in isolation an also include other landmarks as well (e.g., landmarks 9 and 5 on L4; landmarks 1 and 2 on L1; Figs. 7, 8, 9, 10).

The matrix permutation tests (Table 11) indicated that the VCV matrices of karyotypes and crosses were never significantly correlated with the VCV matrices of FA and measurement error. The high correlation between the VCV matrices of the interindividual and FA effects for females at 18°C was basically due to the (micro-) environmental component. Also notice that all correlations between the VCV matrices of karyotype and FA effects were close to zero or even negative, which clearly suggests that this genetic component of canalization is unrelated to DS.

In addition, the PC1s of karyotypes and FA were nearly at right angles (18°C: females *α *= 85.8°, males *α *= 77.3°; 23°C: females *α *= 75.6°, males *α *= 78.5°). The only matches were between PC2 of karyotypes and PC1 of FA for females at 18°C (*α *= 13.0°) and males at 23°C (*α *= 31.2°). The PC1s of crosses and FA were also poorly correlated; the only exception being females a 23°C (*α *= 43.3°). These results clearly support the hypothesis that genetic canalization and DS are not functionally the same mechanism.

On the other hand, all observed angles involving PC1s between "replicated genotypes" (i.e. the between-fly component) and FA were relatively small and highly significant (18°C: females *α *= 20.1°, males *α *= 15.9° ; 23°C: females *α *= 22.7°, males *α *= 36.7°). (Results were qualitatively the same for all observed angles involving PC1s of the between-fly and measurement error covariance matrices; results not shown.) Together with the overall comparisons of the covariance matrices (Table 11), these results indicate that (micro-) environmental canalization and DS share underlying regulatory mechanisms but are not identical. There was not a complete congruence as PC1 of FA accounted for most part of the variation, while PC1 of between-fly variation usually explained less than 50% of the total variance (Figs. 7, 8, 9, 10).

To conclude, the theoretical lower limit for (micro-) environmental canalization (i.e., the environmental variance among genetically identical individuals) would be FA because the two sides share the same genome (barring unusual somatic mutation or somatic recombination) and nearly the same environment, so differences between sides are likely to be small. Under stabilizing selection this lower limit is obviously associated with higher fitness. However, this "canalization limit" would hardly ever be observed because of unavoidable additional sources of environmental variance (e.g. variation between vials, the position of the pupae in a vial, etc.). A similar logic than the one used in this work has been applied to distinguish between intrinsic and extrinsic stochastic variation in gene expression: intrinsic noise can be separated by contrasting the levels of gene expression in a construct with two identically regulated but fluorescently distinguishable *gpf *genes in the *Escherichia coli *chromosome, whereas extrinsic noise is inferred by the correlated variation between the two copies in the same environment [52,53].

## Conclusions

This study applied the methods of geometric morphometrics in the context of quantitative genetics of wing form variation using isochromosomal lines of *D. subobscura*. The main findings can be summarized as follows: (i) for the analysis of overall size, DS was positively correlated with levels of heterozygosity (i.e., inbred *vs*. outbred homokaryotypes) and development at the optimal temperature; however, no positive association was found between DS and chromosomal heterozygosity in outbred crosses; (ii) there was detectable genetic variation (mainly for overall shape) for the directional component of morphological asymmetry (i.e., DA) but not for FA, which likely reflects variation due to stochasticity in development; (iii) for analyses of shape, the patterns of covariation for FA and measurement error were highly concordant in all samples, which also provides strong reasons to conclude that FA is generated by random perturbations of developmental processes (obviously, this does not imply that DS is independent of the genetic background: wing shape FA was found to be higher in inbred females at 18°C when compared to their outbred homokaryotypic counterparts); (iv) the inter- and intraindividual variation patterns were generally poorly correlated, which supports the hypothesis that canalization and DS are distinct mechanisms; however, (v) the patterns of variation due to the (micro-) environmental component of canalization (i.e., the among-fly special environmental effects covariances) were quite similar to those observed for FA; (vi) the lack of a significant within-group correlation between the VCV matrices associated with the interindividual genetic components of canalization and FA, as well as the low similarity between the corresponding vectors describing variation of landmark position, strongly suggest that genetic and environmental canalization are not similar mechanisms.

In addition, (vii) a discrepancy between sexes was observed in some situations; e.g. overall size FA increased with inbreeding and (sub-optimal) temperature effects mainly in females, and the allometric effect on wing shape at both experimental temperatures was similar in females but not in males. It is also interesting to note here that wing size (measured as WL; Fig. 12) clines for *D. subobscura *developed in North America after ~20 years since colonization, but males were clearly lagging behind females [54]. What is not obvious, however, is why there is a difference between the sexes.

It has been suggested that a relationship between canalization and DS could only reflect a common underlying association between character and fitness [55,56]. Those traits under strong stabilizing selection may not be genetically canalized and the major source of selective pressure for canalization can result from the benefits gained by buffering the effects of environmental perturbations [4,10]. The strongest evidence in favor of this hypothesis comes from the well-known genotype-phenotype mapping of RNA folding. Conservation of RNA secondary structure is under strong selection, and low structural plasticity is achieved through increasing the thermodynamic independence of any one structural component from the remaining structure [57]. Likewise, the flux summation theorem developed in the field of metabolic control analysis implies, if true, that phenotypic robustness is an inevitable outcome of the underlying metabolism and not a result of evolution (see [58]).

However, it is still an open question whether or not natural wing shape changes in *Drosophila *are adaptive. There are no consistent patterns between latitude and wing shape (e.g. [30]), contrarily to what happens for size-related traits where world-wide latitudinal clines are found with genetically larger individuals derived from higher latitudes (e.g. [30,59]). Many genes with small additive effects on features of wing shape are dispersed along the *Drosophila *genome (e.g. [60,61]), and we have shown here that the wing shape index L1/WL appears to be a purely additive trait since heterokaryotypes were always intermediate to their homokaryotypic counterparts. The wing shape cline in North America colonizing populations of *D. subobscura *[30] can be largely accounted for parallel latitudinal clines in chromosomal gene arrangements [32,33], and the small shifts of (e.g.) the anterior and posterior crossveins in relation to karyotype variation (Figs. 7, 8, 9, 10; notice that the plotted joint variation in landmark positions is an exaggeration of the actual variation in the data set) are difficult to link with any adaptive response to a better flight capacity. Actually, we lack even hypothetical functional explanations for subtle shape variation: Gilchrist et al. [54] speculated that wing shape variation in *D. subobscura *may simply represent drift around an optimum. Our present results (points (v) and (vi) above) give some credence to that conjecture. Genetic canalization on wing shape does not seem to arise as a by-product of environmental canalization and, therefore, canalization is not a single mechanism to buffer any source of variation as has been suggested [10].

According to Graham et al. [62] the classical linear theory of DS can successfully account for both normally distributed error distributions and leptokurtic distributions caused by the admixture of individuals having different levels of DS, but cannot account for transitions between FA and DA. We have previously suggested, however, that a transition from "ideal" FA (i.e., a normal distribution of left – right-side scores whose mean is zero) to a distribution showing DA could be made entirely compatible with what it is already known from classical quantitative genetics [38]. Shifts between asymmetry types (FA, DA and antisymmetry) have been reported to happen along a species distribution range [63], but unless the genetic component can be partitioned out the variation in left-right differences cannot be assumed to describe DS. From the results of outbred crosses reared at 18°C (Table 5, 6) it is possible to test here for the congruence between patterns of morphological variation with respect to the variation attributable to FA (i.e. the within-fly environmental component of the interaction term) and that attributable to genetic variation for DA (the within-fly genetic component due to crosses in karyotypes of the interaction term). The corresponding VCV matrices were highly correlated for females (MC = 0.914, *P*_(permutation) _= 0.0001) but not for males (MC = 0.064, *P*_(permutation) _= 0.485). The angles between the PC1s also reflect this discrepancy between samples (females *α *= 8.6° ; males *α *= 45.9°). When considered together, these results clearly suggest that FA and genetic variation for DA may or may not be functionally linked.

## Methods

### Extraction of O chromosomes and fly handling

A large number of *D. subobscura *isochromosomal lines for the O chromosome in an otherwise homogeneous genetic background were derived from an outbred stock collected at Puerto Montt (Chile; 41° 28' S) in November 1999 as previously indicated [38]. Briefly, wild-type males were individually crossed to three or four virgin females from the highly homogeneous *ch-cu *marker strain, which is homozygous for the morphological recessive markers on the O chromosome *cherry *eyes (*ch*) and *curled *wings (*cu*) and fixed for the gene arrangement O_3+4_. A single  / O_ + *cu *+ *ch *_male from the offspring was backcrossed to *ch-cu *females, and its arrangement on the wild-type chromosome was identified after four generations of backcrosses. Followed by at least another backcross to *ch-cu *females, a single male from each line carrying the wild chromosome was crossed to two virgin females from the *Va/Ba *balanced marker stock. This strain was derived from the *ch-cu *stock and carries the dominant lethal genes *Varicose *(*Va*) and *Bare *(*Ba*) on the O chromosome. The isochromosomal lines were established from the final crosses ♀♀ O_*Va ch *+ *cu *_/  × ♂♂ O_*Va ch *+ *cu *_/ . All lines used here had a quasi-normal viability according to the recorded proportions of wild-type flies raised in the final crosses to obtain the isochromosomal lines [38]. The lines were kept at 18°C (12:12 light/dark cycle) in 130-mL bottles with low adult density to standardize the rearing conditions before egg collections.

As previously indicated the experimental flies were obtained from 54 crosses. Reciprocal crosses were made for all outbred combinations by mating one-week virgin females and males. After three days the males were discarded and equal numbers of females from each reciprocal cross were placed together in a plastic chamber with a spoon containing non-nutritive agar with a generous smear of live yeast for egg collections. To standardize the experimental conditions, eggs from the inbred (isogenic) crosses were also obtained in a similar way; namely, after mating the flies in bottles and transferring the females to plastic chambers. Eggs were placed in six 2 × 8 cm vials with 6 mL of food (26 eggs/vial); three vials were kept at 18°C (optimal temperature) and the other three at 23°C (sub-optimal temperature). Within each experimental temperature the vials were randomly placed on the same incubator shelf. As a result, the total experiment consisted of 324 vials (162 vials at each experimental temperature), and all eggs were sampled on the same day. Emerging flies (not less than 2 or 3 days old) were stored in Eppendorf tubes with a 3:1 mixture of alcohol and glycerol at 4°C before wing measurements.

All fly handling was done at room temperature using CO_2 _anesthesia on flies not less than 6 h after eclosion.

### Wing measurements

Two randomly sampled females and males emerged from each vial were used for morphometric analyses. Both wings were removed from each fly and fixed in DPX under coverslips on microscope slides. Bitmap images were captured with a video camera (Sony CCD-Iris, Tokyo, Japan) connected to a PC computer with MGI VideoWave software and mounted on a compound microscope (Zeiss Axioskop, Jena, Germany), using a 2.5 × objective. Calibration of the optical system was checked at each session. The images were stored on a Dell Workstation PWS350. To quantify and minimize measurement error all wings were digitized two times at different sessions as follows: images of both the left and right wings were captured during a given session and after an entire round on all individuals the same process was repeated again. A similar procedure was also used to record the *x *and *y *coordinates of 13 morphological landmarks (i.e., labeled geometric points located at the intersections of wing veins or at sites where veins reach the wing margin; Fig. 12) by using the Scion Image for Windows software [64]. Therefore, the process we used guaranteed that the observer was blind with respect to the results from previous measurements.

### Analysis of wing size and shape

Geometric morphometrics precisely separates morphological variation (i.e., variation in form) into size and shape components [21,22]. Size is a one-dimensional trait and the measure most widely used in geometric morphometrics is centroid size (CS), computed here in a normalized form as the square root of the sum of squared Euclidian distances between each landmark to the centroid (center of gravity) of all landmarks divided by the square root of the number of landmarks. Individual size is therefore represented by four scalars, one for each side and session.

The shape of an original configuration of landmarks is the geometrical information that is invariant to uniform scaling (variation in size), translation (differences in position), and rotation (differences in orientation). In contrast to size, shape is an inherently multidimensional space and we used Procrustes superimposition to characterize shape variation. This method allows comparing configurations of landmarks by optimally superimposing (according to a least-squares criterion) homologous landmarks in two or more specimens to achieve an overall best fit [65].Because the data set included both left and right wings (i.e., we are dealing with "matching symmetry" [66,67]) our analyses also removed differences due to reflection by changing the sign of the *x *coordinate of every landmark for configurations from the right side. The reflection, scaling, and superposition steps were performed for all wings within each cross and temperature simultaneously, which allows contrasting wing shapes between different lines or crosses. The final iteration to minimize the sum of the squared distances between the landmarks of all wings in the sample was done without additional scaling and, consequently, we performed a partial Procrustes fit according to Dryden and Mardia [22]. Given the small amounts of shape variation in this analysis rescaling the coordinates of each configuration by the scaling option 1/cos(*ρ*) [65] would have negligible effects on the results.

The landmark coordinates after Procrustes superimposition are amenable to standard multivariate analyses. However, it is important to remember that the removal of size, position (in two dimensions), and orientation reduces the dimensional space to 2*p *– 4, where *p *is the number of landmarks [22]. Thus, for the present study of 13 landmarks, with 2 coordinates each, the shape dimension is 22. Sums of squares and cross-products (SSCP) matrices are therefore not full-ranked, and the degrees of freedom need to be adjusted. There are three alternative ways of avoiding these difficulties [22,67]: (i) to omit, after Procrustes superimposition of the complete configurations, the coordinates of any two landmarks; (ii) to retain 22 PC scores from the covariance matrix of the data set; (iii) to slightly modify the multivariate statistics (see below) by using the Moore-Penrose generalized inverse of the SSCP matrices so they can tolerate singular matrices, and compute the product of nonzero eigenvalues instead of the determinant of SSCP matrices. We have used here the second scheme.

### Experimental design and asymmetry analysis

Quantitative genetic studies of directional and fluctuating asymmetry obviously require measures from individuals that can be grouped into families or independent lines. Our final data set was a fully balanced design, comprising 54 crosses × 3 vials per cross × 2 females per vial × 2 males per vial × 2 sides per fly × 2 measurements per wing × 2 temperatures = 5,184 wing landmark configurations in total. Within each sex and temperature, least-squares (ANOVA) estimates of variance components (i.e. CS) can be easily obtained from the linear model:

*Y*_*ijkl *_= *μ *+ *κ*_*i *_+ *l*_*j*(*i*) _+ *ν*_*k*(*ji*) _+ *ε*_*l*(*kji*)_,

where *μ *is the overall grand mean, *κ*_*i *_is the effect of the *i*th karyotype, *l*_*j*(*i*) _is the random effect of the *j*th cross within karyotype *i*, *ν*_*k*(*ji*) _is the random effect of the *k*th vial within cross *j *and karyotype *i*, and *ε*_*l*(*kji*) _is the residual error associated with the trait (i.e. the individual means computed from both sides and the two replicated measurements per side) of the *l*th individual within vial *k*, cross *j*, and karyotype *i*. Since there was no genetic variation within crosses, the residual error provides an estimate of the total special environmental effects variance (i.e. ). Variation among the three replicated vials was generally negligible (results not shown) and, therefore, we have conveniently reduced the previous model to a two-level nested ANOVA after grouping flies across vials.

To first partition the total phenotypic variation into interindividual, intraindividual and measurement error components, we used the conventional mixed model, two-way ANOVA (or its MANOVA generalization; see below) for the study of left-right asymmetries [39]. In this ANOVA the main random effect of individuals stands for phenotypic variation in the trait (i.e. CS), the main fixed effect of body side is for directional asymmetry (DA) and tests whether or no the signed differences between the left and right wings [designated as ()] have a mean of zero, the interaction term is a measure of fluctuating asymmetry (the variation in left-right differences among individuals) provided that there is no genetic variation for DA [51], and the error term gives an estimate of the measurement error. The two-level nested ANOVA can be straightforwardly subsumed within the two-way ANOVA.

We now digress slightly to point out some inconsistencies in the literature on what is the appropriate error term to test for the "interindividual" effect in the mixed model, two-way ANOVA (either the individual × side interaction effect or the measurement error [13,15,68,69]). Interindividual variation, even if of no general interest in most studies of asymmetry, comprises here genetic components ("karyotype" plus "crosses within karyotypes") and special environmental effects variance (; there is no genetic variance within crosses). An estimated of the among-fly special environmental effects variance (i.e. ) is therefore obtained by subtracting the individual × side interaction effect (which includes  plus measurement error) as the appropriate error term. However, when genetic variation for DA is present the unbiased within-fly special environmental effects variance (i.e. FA) is estimated after partitioning the individual × side interaction effect into its causal components [51].

As pointed out by Klingenberg et al. [67] it is fairly straightforward to extent the preceding ANOVA approach to a full two-factor MANOVA to analyze wing shape asymmetry since all effects are computed from averages or contrasts in the same shape space. Recall that the traces of the corresponding SSCP matrices are just the sum of squares in the Procrustes ANOVA as implemented by Klingenberg and McIntyre [13], but this ANOVA is based on an isotropic model (i.e., it assumes that there is an equal amount of non-directional variation at each landmark [70]) that is not generally correct for any real data. Covariance (VCV) matrices for each effect in the MANOVA were calculated as a simple multivariate extension of the two-way ANOVA. Thus, the SSCP matrices were divided by the appropriate degrees of freedom, and effects were separated according to the expected mean squares in the ANOVA by subtracting the interaction covariance (VCV) matrix from the interindividual VCV matrix, and the error VCV matrix from the interaction one. Therefore, for (e.g.) outbred crosses the interindividual covariance components were calculated as  and the covariance components of FA as , were SSCP_I _is the interindividual SSCP matrix, SSCP_IS _is the interaction SSCP matrix, and SSCP_ME _is the measurement error SSCP matrix.

The SSCP_I _matrix was further partitioned into among-karyotype SSCP_K _matrix, among-cross within karyotype SSCP_C⊂K _matrix, and the residual SSCP_*e *_matrix corresponding to the special environmental effects. As a result, genetic effects for overall wing shape were separated from special environmental effects according to the expected means squares in the two-level nested ANOVA. Therefore, for (e.g.) outbred crosses the karyotype covariance components were calculated as  (remind that the entries in the SSCP_I _matrix are equal to those computed from individual means times twice the number of independent measurements per wing), the cross covariance components as , and the among-fly special environmental effects covariance components as .

Similarly, genetic effects for DA can be investigated after partitioning the SSCP_IS _matrix into their causal components [51].

### Morphological patterns of variation

Within each sex and temperature, principal component analyses [41] of the VCV matrices were performed for each source of variation with the purpose of describing the landmark displacements corresponding to each emerging principal component (PC), and also to test for the congruence of these displacements between effects. This technique extracts new shape variables (PCs) which successively account for the maximal amount of shape variation and contain information on how the variables relate to each other. The PCs form an orthonormal set of vectors (i.e., the inner product  for i ≠ j,  for i = j; superscript 'denotes transposition) in an n-dimensional space.

Correlations between corresponding VCV matrices were computed from the upper triangular part (diagonal entries were included) since covariance matrices are symmetrical, and statistical significance was assessed using permutation tests designed to maintain the associations between pairs of *x*- and *y*-coordinates (i.e., by permuting pairs of rows and columns [13,15]); otherwise the null hypothesis would imply the complete absence of all geometric structure. The permutation procedure was carried out 10,000 times. Correlative patterns of whole shape variation are difficult to interpret: a significant correlation would suggest a real congruence, but a weak congruence does not imply a significant correlation.

A second test examined the congruence of the landmark displacements corresponding to each emergent PC for the different effects within groups. Because the PCs correspond to directions in the multivariate shape space, correlations can be obtained by angular comparisons of component vectors. Statistical significance of these correlations was then assessed by comparing those observed values to a null distribution of absolute angles between 100,000 pairs of 22-dimensional random vectors [71]. The 0.1% and 0.001% quantiles of the resulting distribution were 50.3° and 41.6°, respectively.

### Antisymmetry and allometric effects

The occurrence of antisymmetry (AS: a bimodal distribution of signed () [39]) for centroid size was investigated within each sample using the Lilliefors (Kolmogorov-Smirnov) test for the composite hypothesis of normality [69]. The independence between size and size FA within each sample was assessed by a linear regression of unsigned () against mean centroid size .

Scatter plots of left-right differences for each landmark after Procrustes superimposition were visually checked to see whether or not there was evidence for clustering of these vectors that would have argue for the occurrence of AS [13,15]. No indication of AS was detected. Finally, to test for size effects on shape asymmetry within each sample we used multivariate regression of vectors of both signed and "unsigned" shape asymmetries onto mean centroid size [13]. Shape asymmetries were not related to size (*P*-values > 0.10) and, therefore, no size corrections were necessary.

### Computer software for statistical analysis

The computer programs used for statistical data analyses were MATLAB (V.6. [72]) together with the collection of tools supplied by the Statistics Toolbox (V.3. [73]). Some helpful functions in morphometrics from the MATLAB toolboxes Res5 and Res6 developed by R. E. Strauss [74] were also used. Results (e.g., derivation of SSCP matrices) were checked with the statistical software packages STATISTICA V.6 [75] and SPSS V.11 [76].

## Authors' contributions

MS conceived the study, carried out extraction of O chromosomes, experimental crosses, egg collections, statistical analyses, and drafted the final manuscript. PFI carried out extraction of O chromosomes, experimental crosses, egg collections, wing measurements, and preliminary statistical analyses and drafts of results. WC read all salivary gland squashes for gene arrangement identification and mounted the wings on microscope slides. All authors read and approved the final manuscript.
